# Genetics of Marine Organisms Associated with Human Health

**DOI:** 10.3390/md18110548

**Published:** 2020-11-02

**Authors:** Maja Herak Bosnar, Helena Ćetković, Matija Harcet

**Affiliations:** 1 Division of Molecular Medicine, Ruđer Bošković Institute, Bijenička 54, 10000 Zagreb, Croatia; 2 Division of Molecular Biology, Ruđer Bošković Institute, Bijenička 54, 10000 Zagreb, Croatia

The aim of this special issue was to provide insight into the field of research on genetics and genomics of marine organisms linked with human health. Marine habitats harbour a large variety of organisms that belong to diverse taxa; from bacteria and unicellular eukaryotes to fungi, animals, and plants. Although we have only started to understand the diversity and structure of marine communities, it is clear that numerous marine species have or might have an impact on human health. Some are a source of natural products with potential or actual medical applications, others are toxic and harmful to humans, and some are used in biomedical research to help understand the molecular basis of human diseases. New molecular genetics and genomic methods provide powerful and ever more indispensable tools for studying marine organisms and all aspects of their influence on human health. Herein, we present work using the latest research, which mostly uses genomics, to tackle the questions related with the topic of the issue.

This Special Issue contains eight research articles on different topics related to genetics and genomics of marine organisms associated with human health. In the following sections, we would like to provide a short overview of what the reader will find in this Special Issue.

Jia and colleagues report a high-quality genome with a relatively complete gene set for the Chinese white dolphin (*Sousa chinensis*). They discovered that the expansion of certain gene families, especially the increase in immune and sensory genes, could shed some light on the molecular mechanisms for adaptation to the nearshore environments. Furthermore, they observed a serious bottleneck in the demographic population history of Chinese white dolphin before the Mindel glaciation (about 350,000 years ago). In addition, a comparative genomic survey on antihypertensive peptides (AHTPs) increases knowledge about the potential of mammal proteins for development of antihypertensive peptides. Genome assembly of Chinese White Dolphin provides a genetic resource for other studies on adaptive ecology, conservation biology of cetaceans and development of marine peptide-based drugs for treatment of various human cardiovascular diseases [1].

Wang and colleagues provide a high-quality genome of the giant grouper (*Epinephelus lanceolatus*). Giant groupers are of economic importance in marine aquaculture for their rapid growth, but unfortunately bacterial and viral diseases have become the main threats to the grouper industry. The authors identified antimicrobial peptide (AMP) genes systematically in groupers and, therefore, support antimicrobial research and possibly provide suggestions for therapy. These genomics resources will help in the effort against infectious diseases in the giant grouper industry [2].

Chen and colleagues generated a high-quality genome assembly of the Japanese eel (*Anguilla japonica*). They also performed phylogenetic and synteny analyses, as well as variation determination of 24 tbx4 sequences in 22 representative teleost species. They determined, for the first time, an inversion of a special tbx4 gene cluster that is potentially correlated with the evolutionary development of teleost fin spines, which may benefit the development of novel marine drugs [3].

Bian and colleagues sequenced and assembled a draft genome of the blue tilapia (*Oreochromis aureus*) for the first time. They performed a series of genomic analyses related to biology and immunology (such as antimicrobial peptides (AMPs)) of the blue tilapia and genetic comparisons with its close relative, the Nile tilapia (*O. niloticus*). They also predicted 23,117 protein-coding genes in the blue tilapia genome. Comparisons of predicted antimicrobial peptides between the blue tilapia and its close relative, the Nile tilapia, proved that these immunological genes are highly similar with a genome-wide scattering distribution. Given that the blue tilapia has an important economic value, its genome resource will build a valuable platform for further biomedical research and practical molecular breeding of tilapias [4].

In their article, Gill and Kumara present a novel, alternative approach for testing developmental neurotoxicity. This strategy is rapid and economically feasible with an acceptable predictive capacity, as the previously used models do not necessarily reflect physiological differentiation processes involved in neurogenesis, astrocytogenesis and oligodendrocytogenesis. They chose rat neuronal stem cells (rNSC) as a model system, which will enable usage of dose ranges that are measured in humans in a high-throughput mode, testing multiple chemicals simultaneously at a much lower cost. As a test chemical, the authors used domoic acid (DA) produced in blooms of different marine species such as species of *Pseudo-nitzschia* or red alga and ochratoxin A, a mycotoxin produced in *Aspergilli* and *Penicilia* molds. The test showed that both non-cytotoxic and cytotoxic concentrations of DA and OTA reduced the differentiation of rNSC into astrocytes, neurons and oligodendrocytes, which indicates that low, non-cytotoxic concentrations of these compounds interfere with the differentiation of rNCS. This proves that the rNSC system is beneficial in these kind of investigations [5].

The work of Perina et al. reports new findings on one of the poorly studied members of the NME/NDPK family of genes/proteins. Nucleoside diphosphate kinase are a family of enzymes that transfer the terminal phosphate from NTPs (mostly ATP) to other d(NDPs). All of the members of the NME/NDPK family possess the NDK domain but only NME1–NME4 exhibit NDPK activity. The NME/NDPKs have been linked to many human diseases such as metastasis regulation and cardiac diseases. The authors report on the NME5 homologue from an early branching eukaryotic lineage, the red alga *Chondrus crispus*. The ancestral type protein is, unlike its human homologue, a functional multimeric NDPK with high affinity to various types of DNA. Its overexpression inhibits cell proliferation and the anchorage-independent growth of cells in soft agar, but has no impact on apoptosis. It seems that the ancestral gene has changed during eukaryotic evolution, probably together with its protein function [6].

Seahorses are well known for their special shape and male pregnancy but have also been an important resource for traditional Chinese medicine. Five seahorse species have been applied in the production of these medicines, especially the lined seahorse (*Hippocampus erectus*). Large-scale aquaculture of seahorses has lately been seriously limited due to fatal diseases caused by external pathogens such as vibrio or ciliate. The antibiotic applied often causes drug resistance and residual drug issues; therefore, finding novel antibacterial alternatives to secure seahorse and other aquaculture populations is urgent. In their work, Chen and coworkers analyzed putative antimicrobial peptides (AMP) from the previously studied genome and transcriptome studies of the lined seahorse in a high-throughput manner and checked their transcriptional changes in various tissues during development. Furthermore, differential expression between male and female individuals was also analyzed for potential sexual variances and specificity. The data provides an overview of AMPs in the popular lined seahorse, which is a solid foundation for further investigation on development of AMP-based fish food and human drugs [7].

Ilamycins are bioactive antimicrobial cyclopeptides derived from marine *Streptomyces*. He et al. report on the functions of four genes of the ilamycin biosynthetic gene cluster (*ila* BCG), including two regulatory genes, a postive *ilaA* and a negative *ilaB* regulatory gene together with two transporter genes (*ila J* and *ilaK*). The authors characterized the mentioned genes, used them to construct high-yield strains and expressed the biosynthetic gene cluster of ilamycin in *Streptomyces coelicolor* 1152. These results lay a rather good foundation for further exploration but also provide basic genetic elements for synthetic biology [8]. 

As guest editors, we are grateful to all the authors who contributed their excellent results to the special issue, all the reviewers who carefully evaluated the submitted manuscripts, and the editorial boards of Marine Drugs, and Vincent Di, Assistant Editor, for their support and kind help.
